# Accumulation and trafficking of zinc oxide nanoparticles in an invertebrate model, *Bombyx mori*, with insights on their effects on immuno-competent cells

**DOI:** 10.1038/s41598-020-58526-1

**Published:** 2020-01-31

**Authors:** Ashiq Hussain Mir, Ayesha Qamar, Ishana Qadir, Alim H. Naqvi, Rizwana Begum

**Affiliations:** 1 Section of Entomology, Department of Zoology, Aligarh Muslim University, Aligarh, 202002 Uttar Pradesh India; 2 Interdisciplinary Nanotechnology Centre, Aligarh Muslim University, Aligarh, 202002 Uttar Pradesh India; 3 Department of Zoology, School of Bioengineering and Biosciences, Lovely Professional University, Phagwara, 144411 Punjab India

**Keywords:** Zoology, Animal physiology

## Abstract

Zinc oxide nanoparticles (ZnO NPs) are used in many applications; however, their interactions with cells, immune cells in particular, and potential health risk(s) are not fully known. In this manuscript, we have demonstrated the potential of ZnO NPs to cross the gut barrier in an invertebrate model, *Bombyx mori*, and that they can reach the hemolymph where they interact with and/or are taken up by immune-competent cells resulting in various toxic responses like decline in hemocyte viability, ROS generation, morphological alterations, apoptotic cell death, *etc*. Exposure to these NPs also resulted in alteration of hemocyte dynamics including an immediate increase in THC, possibly due to the release of these hemocytes either from enhanced rate of cell divisions or from attached hemocyte populations, and decline in percentage of prohemocytes and increase in percentage of two professional phagocytes, *i.e*., granulocytes and plasmatocytes, possibly due to the differentiation of prohemocytes into phagocytes in response to a perceived immune challenge posed by these NPs. Taken together, our data suggest that ZnO NPs have the potential to cross gut barrier and cause various toxic effects that could reverse and the insects could return to normal physiological states as there is restoration and repair of various systems and their affected pathways following the clearance of these NPs from the insect body. Our study also indicates that *B. mori* has the potential to serve as an effective alternate animal model for biosafety, environmental monitoring and screening of NPs, particularly to evaluate their interactions with invertebrate immune system.

## Introduction

The nanotechnology has developed rapidly in the present world and engineered nanoparticles (NPs) have found tremendous applications in diverse products like cosmetics, pharmaceuticals, food industry products, paints, electronics, clothing, *etc*.^1–3^ Such large-scale use of nanoparticles has raised concerns with regard to their impacts on human health, biodiversity, environment and ecosystems; more so because of their small dimensions and other unique properties. Since precise cellular mechanism(s) of nanoparticle interaction with biological systems are mostly unknown, nanotoxicology demands comprehensive assessments of various interactions between NPs and biological systems based upon their various exposure routes. The gravity of the environmental concerns regarding the use and disposal of NPs has attracted a plethora of studies to assess their toxicity, both *in vivo* and *in vitro*^4^. Exposure to various NPs has been reported to cause oxidative stress, cytotoxicity, DNA damage, apoptosis, necrosis, genotoxicity, aberrant mitochondrial function and reduced photosynthesis in different cell lines^5–11^. Various *in vivo* studies in a wide spectrum of organisms have linked NP exposure to various toxic effects like developmental abnormalities in zebra fish embryos^12,13^, decline in vertebrate lung function^14^, interaction with sea urchin immune cells^15^, increased mutagenesis in *Drosophila*^16^, hepatic toxicity in mice^17^ and reduced survival in fish and *Daphina*^18^.

Zinc oxide NPs, owing to their UV light absorption, catalytic properties, antimicrobial potential, semiconducting and magnetic properties, *etc*., have found tremendous application in rubber manufacture, food additives, pigments, cosmetics, medicine, electronics, *etc*. Recently, ZnO NPs have attracted much attention, possibly, for their use in cancer therapy as they have been reported to induce selective cancer cell killing^19^. However, the risks associated with their widespread use have come to fore as various studies with diverse animal models have suggested the potential of ZnO NP exposure to generate various toxic effects. It has also been shown that ZnO NPs, after systemic distribution, could reach various organs, and exhibit toxic effects on lungs, liver, kidney, pancreas, spleen, stomach, testis, thymus, brain, heart, blood, *etc*.^20–24^ In addition, *in vitro* cytotoxicities have also been reported in many cells like epidermal cells^25^, macrophages^26^, human lung epithelial cells^27^, *etc*.

Until recently, most of the studies on the potential ZnO NPs toxicity have focused on conventional model systems like mammals, aquatic invertebrates and/or on different types of cell lines. Assessment of the effects of NP exposure in insects, terrestrial insects in particular, has been neglected or has attracted only few detailed studies. Insects, because of their sheer number, diversity and ecological position, are one of the most vital groups of organisms of our environment. Therefore, any perceived threat to any other form of life must be assessed in this vital group of organisms as well. Unlike other stable NPs, ZnO NPs, because of their greater solubility, can lead to more complex interactions. Further, interspecies differences in response to these NPs makes the extrapolation of these results to other forms very difficult.

As there is a growing realization that invertebrate immune system provides an ideal model system for investigating various responses, and subsequent evolution, of immune defenses to both natural and anthropogenic stressors^28–30^. Therefore, evaluating the NP interactions with invertebrate immune system is becoming an essential part of such an assessment. Further, studying the effects of NPs in insects will provide valuable insights not only into the immune system of invertebrates but also into the human immune system as insect and human immune responses display extensive functional similarities in several aspects, e.g., they use similar effectors and receptors, and also possess similar gene expression regulation^31^. It has also been reported that several insect immune proteins demonstrate a high degree of homology to vertebrate proteins, such as insect proteins malvolio and dSR-C1, which are identical to mouse natural resistance associated macrophage protein-1 (NRAMP-1)^32^. The insect phagocytic cells, the granular cells and plasmatocytes, bear surface receptors (e.g., calrecticulin) which are quite similar to the mammalian neutrophil receptors, and these cells, in insects as well as humans, engulf and kill pathogens and use similar proteins (p47 and p67) for the production of superoxide^32,33^. Therefore, the current study was undertaken with the aim to understand the accumulation and trafficking of ZnO NPs with focus on their effects on innate immune system in an invertebrate model system and we hypothesized that ZnO NPs could cross biological barriers and reach blood circulation leading to the impairment of the immune function.

Invertebrates lack an adaptive immunity, and immune response in insects comprises two highly interconnected components, the cellular and the humoral responses^34,35^. The cellular responses mediated by hemocytes involve responses like encapsulation, phagocytosis and nodulation^36^. The humoral defenses rely on soluble effector molecules like anti-microbial peptides, melanin, complement-like proteins, and the products of proteolytic cascades, for instance the phenoloxidase pathway, which immobilize and/or kill pathogens^37^. At least eight hemocyte types have been described in insects: plasmatocytes, prohemocytes, granular cells, crystal cells, coagulocytes, spherulocytes, thrombocytoids and oenocytoids^38^; however, the most of the insects don’t possess all of these types at a time. They are found, mostly, freely circulating in the hemolymph or adhering to various internal organs particularly fat body and digestive tract^32^.

Herein, we employed silkworm, *Bombyx mori*, as the model system, which has the potential to serve as an efficient alternative for *in vivo* as well as *in vitro* research, particularly because the unconventional models are thought to be fundamental to filling various knowledge gaps that the well-established models leave in the elucidation of complex mechanisms such as immunity, stress response, cell death, *etc*. Furthermore, replacing mammals by insects as *in vivo* screening systems can dramatically change the speed with which *in vivo* data on biosafety assessment, biomonitoring, and screening of NPs and other novel material can be generated. Therefore, this study could not only serve to assess various effects of ZnO NPs in insects, which represent an ecologically important group of organisms, but also help in the direction of establishing *B. mori* as an alternate animal model for biosafety and environmental monitoring studies.

## Materials and Methods

### ZnO nanoparticles and their characterization

Zinc oxide NPs were obtained from nanotechnology unit, ZHCET, Aligarh Muslim University. The nanoparticles were characterized via following techniques and used as received.

#### XRD analysis

X-ray diffraction (XRD) was performed by XPERT-PRO XRD system (Rigaku Corporation, Tokyo, Japan) operating at 45 kV. Dried ZnO particles were deposited as a randomly oriented powder into a plexiglass sample container and the XRD patterns were recorded between 10**°** and 90**°** angles, with speed of 5.0 deg/min. The average crystallite size ‘D’ of the ZnO-NPs was calculated from the main peak using Scherrer’s equation:

$${\rm{D}}=0.9{\rm{\lambda }}/{\rm{\beta }}\,\cos \,{\rm{\theta }}$$

where, λ is the X-ray wavelength, β is the full width at half maxi-mum (FWHM) of the peak and θ is the peak position^39^.

#### Scanning and transmission electron microscopic analysis

The NP surface morphology was determined using a SEM machine (JEOL- JSM-6510LV, Tokyo, Japan) operated at a voltage of 10 kV. Crystal morphology and particle size of materials was determined by employing Transmission Electron Microscopy (TEM), which was carried out on 100/120 kV TEM (JEOL, Tokyo, Japan) with an accelerating voltage of 200 kV^39^.

#### FTIR

Infrared spectroscopy was used to identify various functional groups as well as adsorbed species and reaction intermediates on the NP surface. FTIR spectra of NPs were obtained with a PerkinElmer FTIR spectrophotometer (in the range 4,000 to 400 cm^−1^) by potassium bromide (KBr) pellet method. Spectroscopic grade KBr was used in the ratio of 1:100 and spectrum was recorded in the diffuse reflectance mode at a resolution of 4 cm^−1^ in KBr pellets^39^.

### Animals

The laboratory stock culture of *B. mori* was established from disease free layings (DFLs) obtained from Regional Sericulture Research Station of Central Silk Board, Dehradun. The eggs, kept in standard rearing condistions of temperature and humidity, hatched out into larvae which were subsequently reared on fresh and clean mulberry leaves at 25 ± 1 °C with a photoperiod of 12 h light and 12 h dark. *B. mori* consists of five larval instars before undergoing pupation inside a silken cocoon. The adults, emerging out of these pupae, were allowed to copulate; and with the laying of eggs completes the life cycle of *B. mori*. Proper rearing conditions were maintained across all developmental forms.

### Administration of nanoparticles

The desired dose of nanoparticles was administered orally to the similar-aged larvae, in the 24 h of the 4^th^ instar (day 2, 4^th^ instar). Briefly, the doses were made by dissolving a desired amount of ZnO NPs in 200 µl of distilled water. Prior to administration, the solution was sonicated for 5 minutes and subsequently applied, as a thin film, on a freshly-cut and clean mulberry leaf, and after the water had dried out, the leaf was used to feed the larvae. Thus the specific dose of nanoparticles was administered only once and to single larvae separately. In case of control group, the leaves used to feed the insects were not charged with nanoparticles. Nanoparticles were applied on a 4 × 4 cm piece of a leaf so that it should be completely consumed before it dries out. After treatment, the larvae were housed in petri dishes and fed routinely; the size of the leaf was no longer a concern. Initially, the larvae were exposed to four different NP doses (0.5 mg, 1 mg, 2 mg and 4 mg per larva) in order to assess the tolerance and immune cell viability and it was observed, as revealed by trypan blue exclusion assay, that the doses of 0.5 mg and 1 mg per insect did not induce significant cell toxicity and the concentration ≥4 mg/insect induced cell toxicity to the levels that we would not be left with sufficient number of cells to work with and, for this reason, the higher concentrations would render further analysis infeasible. Therefore, 2 mg NPs per individual was the dose selected to determine their trafficking and accumulation in various tissues after different time periods and to assess their effect on the innate immune system.

### Measurement of hemocyte viability

After feeding the larvae mulberry leaves charged with different doses (0.5 mg, 1 mg, 2 mg and 4 mg) of NPs, hemocyte viability was determined. Briefly, by cutting one of the larval prolegs was cut with scissors, the hemolymph was subsequently collected in ice-cold tubes. Approximately 0.2 ml of hemolymph was obtained per larva, and 10 µl of each sample were mixed with an equal volume of trypan blue (0.1%) and immediately observed under a microscope. Thereafter, using a cytometer, the number of trypan blue-negative and -positive cells were counted.

### Quantitative estimation of zinc levels in various tissues

The accumulation of NPs was estimated by atomic absorption spectroscopy (AAS). For AAS, the insect material (whole insect, gut, cuticle, ovaries and hemocytes) collected from 10 insects was washed and dried in an oven (at 70 °C for 24 hours). The dried sample was then powdered and 50 mg of the each powdered sample was digested in 5 ml of triacid mixture (HNO_3_:H_2_SO_4_:HClO_4_, 5:1:1) at 80 °C until black fumes turned white and the solution became completely clear^40^. The digest was allowed to cool followed by its dilution with double distilled water and then filtered through Whatman’s No. 42 filter paper. The filtrate was then made up to 5 ml using DDW and then analysed for Zn estimation by an atomic absorption spectrometer (GBC SensAA, Dandenong, Australia). For estimation in hemolymph, wet acid method was employed using 0.5 mL freshly extracted hemolymph.

### Preparation of blood film and light microscopic observations of hemocytes

For microscopic observations of hemocytes, many thin, air dried and Giemsa stained smears were prepared and observed under the Olympus Magnus TR microscope. The best slides were mounted in DPX and stored for characterization and future analysis.

### Scanning electron microscopic (SEM) and energy dispersive X-ray (EDX) studies

SEM was coupled with EDX analysis to obtain information about sample composition and its chemical nature. For SEM, the hemolymph of at least two insects was collected by cutting one of the prolegs of the caterpillar in a cold eppendorff tube containing an equal volume of phosphate buffer (pH = 7.4) and 2.5% gluteraldedyde. The hemolymph was kept undisturbed for fixation for overnight at 0 °C. The fixed cells were centrifuged at 5000 rpm for 10 minutes. The supernatant was discarded and the pellet was washed three times in same buffer (pH = 7.4) and subsequently dehydrated in ethanol grades. Then the pellet was spread, delicately and uniformly, on a clean piece of glass cover slip. The pellet was then coated with gold particles and the observations were made on JSM 650LV scanning electron microscope (JEOL-Japan) fitted with EDX diffractometer.

### Determination of reactive oxygen species (ROS)

The levels of superoxide (O2^−2^) and peroxide (H_2_O_2_) in larval hemocytes was estimated by employing dihydroethidium (DHE; Invitrogen, USA) and 29, 79-dihydrofluorescein diacetate (H_2_DCFDA), respectively, following the methods reported earlier^41^. Briefly, the hemocytes were incubated with respective dyes at the final concentration of 10 mM for 1 h in dark at 24 ± 1 °C. Following a brief PBS washing, cells were finally re-suspended in PBS for analysis. The fluorescence intensity of the oxidized derivatives of two dyes viz., 2-hydroxyethidium for DHE and 29, 79-dichlorofluorescein (DCF) for H2DCFDA was quantified at an excitation/emission wavelength of 535/617 nm and 492/517 nm, respectively. The mean fluorescence intensity was used for the estimation of intracellular ROS level in each sample.

### Determination of apoptotic cell deaths

The induction of apoptosis in hemocytes was analysed by staining them with Annexin V-FITC following the manufacturer’s protocol (Annexin V-FITC apoptosis detection kit) and the previously defined methods^41^. Briefly, the hemocytes were suspended in 500 ml of 1X binding buffer (media binding reagent) followed by addition of Annexin V-FITC (5 ml) and propidium iodide (PI) (10 ml) to the cells. Then the cells were incubated at 24 ± 1 °C for 10 min in dark. Thereafter, FITC signal was detected by FL1 (FITC detector) and Pl by FL2 (phycoerythrin fluorescence detector) at 518 nm 620 nm, respectively. The log of Annexin V-FITC and PI fluorescence was displayed on the X- and Y-axis of the data report respectively. Besides this, the evidence of apoptoic cell death was also obtained from electron microscopic studies.

### Estimation of MDA

Malondialdehyde (MDA) was estimated according to previously mentioned methods. To 500 µl of hemocyte pellet in phosphate buffer (pH 7.4), 300 µl of 30% trichloroacetic acid (TCA), 150 µl of 5 N HCl and 300 µl of 2% w/v 2-thiobarbituric acid (TBA) were added and then the mixture was heated for 15 min at 90 °C. The mixture was centrifuged at 12,000 × *g* for 10 min. Pink colored supernatant was obtained which was measured spectrophotometrically at 532 nm. MDA concentration was measured by using 1,1,3,3-tetraethoxypropane as standard and expressed as percentage of control^42^.

### Estimation of glutathione (GSH)

GSH, a natural antioxidant, level was determined by its reaction with 5, 5′-dithiobis 2-Nitrobenzoic acid (DTNB) using reduced glutathione as standard, at 412 nm wavelength. The processed sample was treated with equal volume of 5% TCA. The mixture was centrifuge at 3,000 rpm for10 min., 0.05 ml of supernatant, 0.1 ml phosphate buffer (pH 8.4), DTNB and 0.05 ml of double distilled water was added. Then absorbance was measured spectrophotometrically at 412 nm within 15 min in Elisa reader (BIOTEK). GSH was expressed as percentage of control^42^.

### Method of differential and total hemocyte count (DHC and THC)

DHC was recorded in permanent stained smears. Many blood smears were made from an individual insect and the best among them were selected for counting, and in each smear at least 200 cells were counted and characterized. The DHC was determined in terms of percentage of different hemocytes in total cells counted.

$$\,{\rm{DHC}}=\frac{Number\,of\,hemocytes\,of\,particular\,type}{Total\,number\,of\,hemocytes}\times 100$$

Total hemocyte counts (THCs) were recorded by applying diluted hemolymph (10 microliter hemolymph added to equal volume of 0.1 M phosphate buffer, pH 7.2) to a Neubauer hemocytometer. THCs were expressed as number of cells per ml of hemolymph.

## Results

### Characterization of ZnO NPs

**Figure 1:**
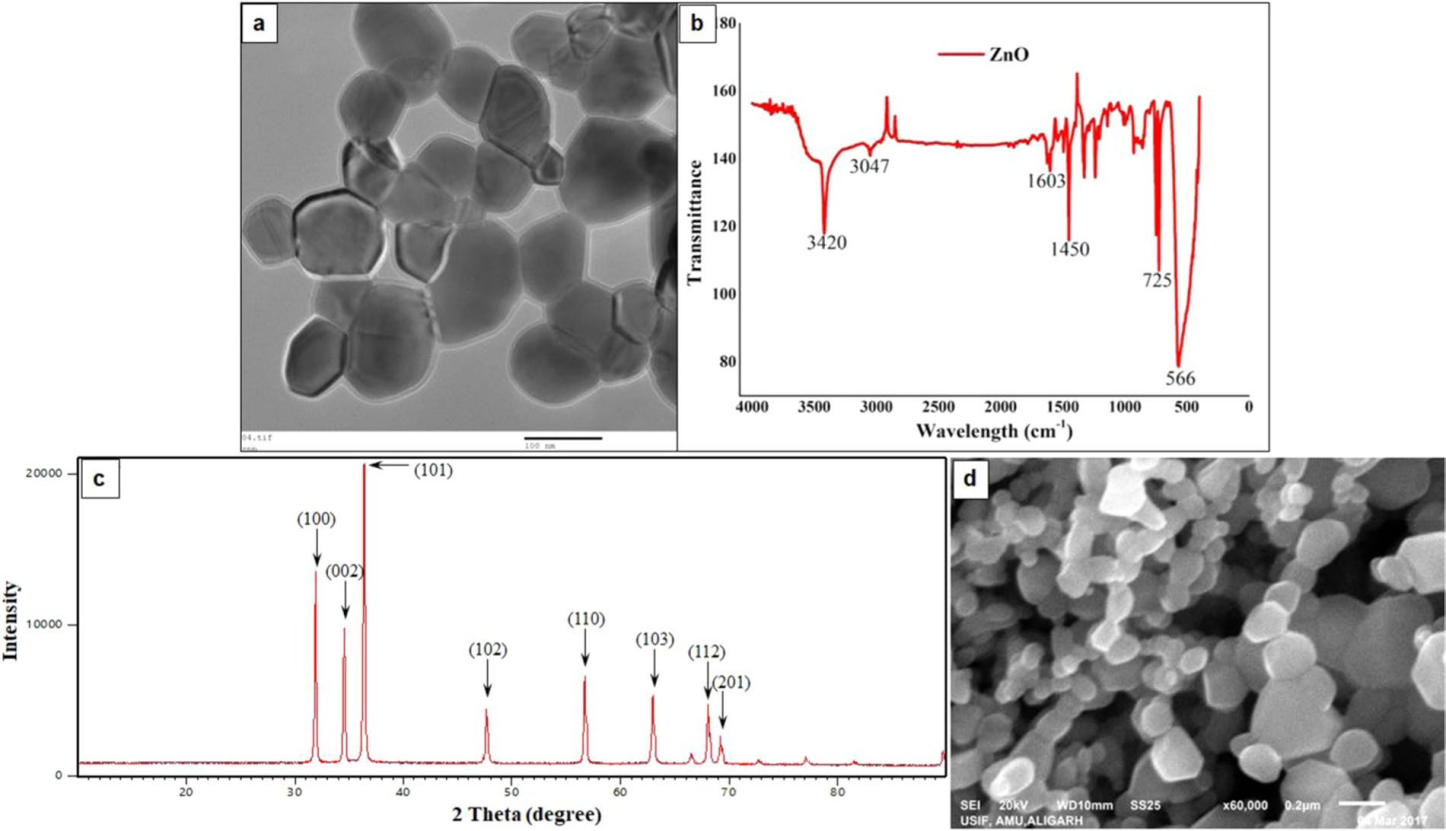
Characterization of ZnO nanoparticles. TEM (**a**) and SEM (**d**) images showing the shape and size of ZnO NPs. In FTIR spectra of ZnO nanoparticles (**b**), the peaks observed at 3420 and 3047 may be due to O-H bond and asymmetric stretching, respectively. Peak at 1603 may be due to O-H bending. Peaks at 725 and 566 are due to stretching mode of ZnO. XRD pattern of ZnO nanoparticles (**c**). Definite line broadening of XRD peaks indicates that the nanomaterial consists of particles in nanoscale range. The peaks at 31.87, 34.52, 36.35, 47.64, 56.69 and 66.47 confirm that the nanomaterial was free of impurities as it doesn’t contain any uncharacteristic peaks other than ZnO peaks.

The NPs were characterized in terms of morphology, size, shape, *etc*. The SEM (Fig. 1d) and TEM images (Fig. 1a) of ZnO NPs exhibit that the majority of the particles were of polygonal shape with smooth surfaces, and the TEM revealed their polyhedron morphology (Fig. 1a). In XRD pattern (Fig. 1c), the peaks at 2θ = 31.67°, 34.31°, 36.14°, 47.40°, 56.52°, 62.73°, 66.28°, 67.91°, 69.03°, and 72.48° were assigned to (100), (002), (101), (102), (110), (103), (200), (112), (201), and (004) of ZnO NPs, indicating that the samples were polycrystalline wurtzite structure (Zincite, JCPDS 5-0664). The absence of any characteristic peaks other than those due to ZnO NP suggests the purity of these NPs. The average TEM diameter of these NPs was found to be 40.06 ± 4.90 nm, which was further supported by the XRD data. The FTIR analysis (Fig. 1b) reveals the presence of various functional groups associated with these NPs, *e.g*., the peaks at 3420 and 3047 may be due to O-H bond and asymmetric stretching, respectively. Whereas, the peak observed at 1603 may be due to O-H bending. Peaks at 725 and 566 are due to stretching mode of ZnO.

### Effects of ZnO exposure on hemocyte viability

There was a significant decline in hemocyte viability following ZnO exposure and this can be attributed to the translocation of these NPs across the gut barrier into the hemolymph and their subsequent interaction and/or uptake by the circulating hemocytes therein. This latter claim is based on our results that have been described in the sections that follow. We also observed a dose dependent decline in hemocyte viability following ZnO exposure (see Supplementary Fig. S1). This study formed the basis to select the exposure dose for further studies. We selected 2 mg/insect as the concentration for our subsequent analysis because the doses milder than this didn’t cause significant cell death, and the higher doses, although, caused significant cell death, but, at the same time, left us with not-so-sufficient number of cells to work with.

### ZnO NP trafficking and interaction with insect immune cells

**Figure 2:**
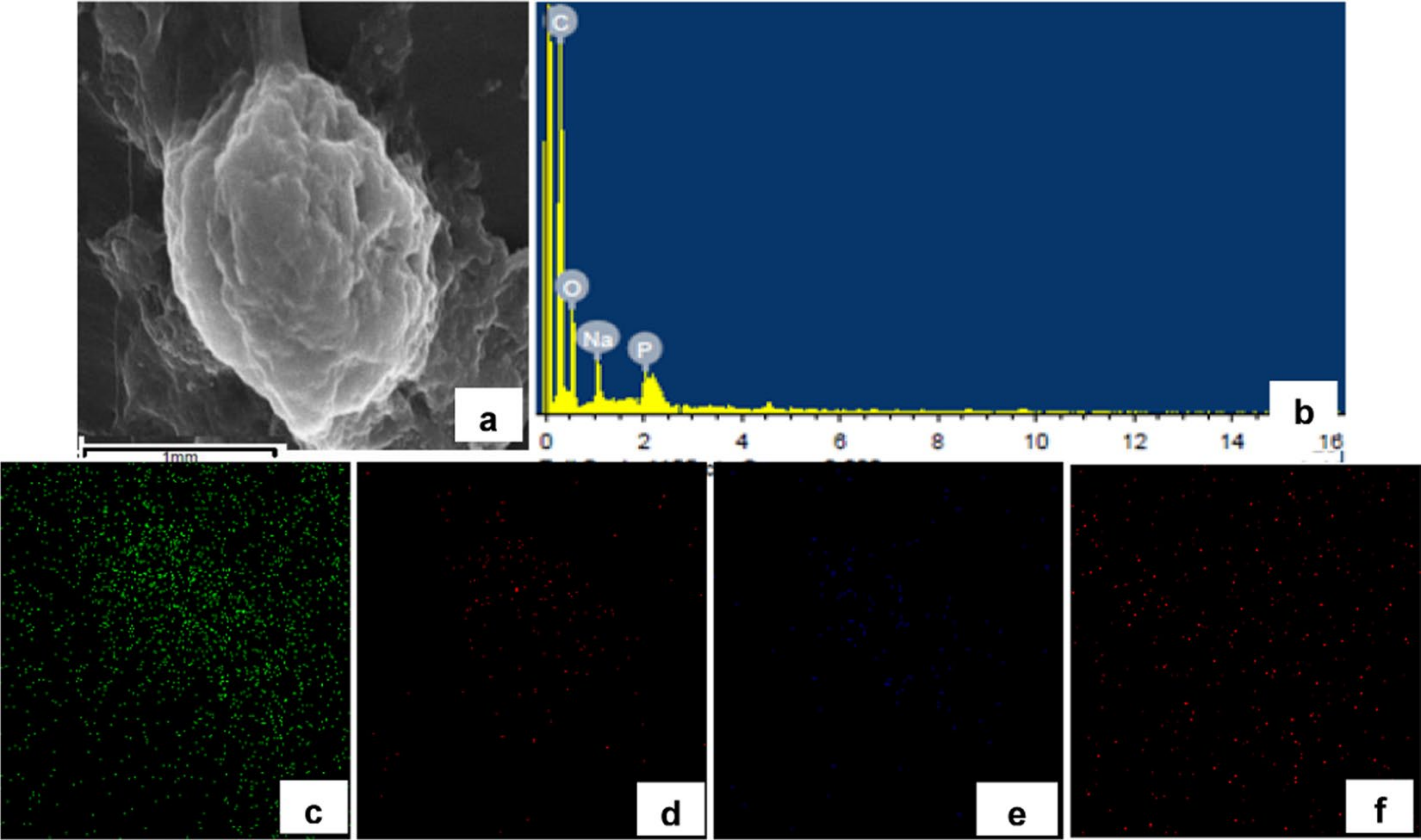
SEM image of a normal hemocyte (plasmatocyte) with corresponding Energy Dispersive X-ray (EDX) analysis confirming the composition and distribution of various elements like Carbon (**c**), Oxygen (**d)**, Sodium (**e**) and Phosphorus (**f**) through spectrum and elemental mapping.

**Figure 3:**
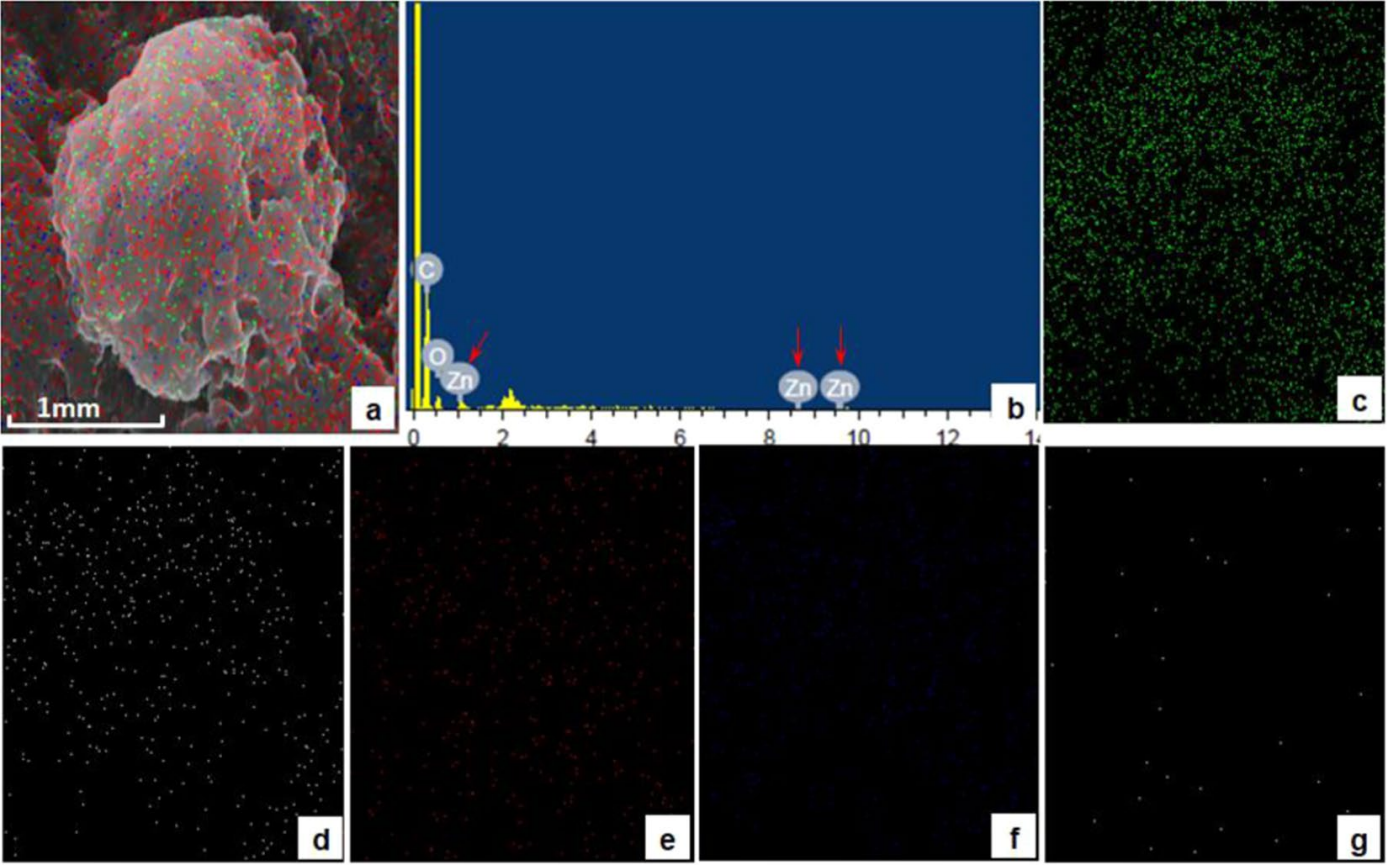
Deposition/presence of various metals on the surface of *B. mori* hemocyte after ZnO NP exposure. Panel a represents SEM image of hemocyte superimposed with Electron Dispersive X-ray (EDX) imaging of all metals present. The characteristic zinc peaks are indicated by the red arrow in panel b. Other panels indicate EDX imaging, on hemocyte SEM micrograph, of Carbon (**c**), Oxygen (**d**), Phosphorus (**e**), Sodium (**f)** and Zinc (**g**).

Thereafter, following the single oral exposure of ZnO NPs, their concentration was determined in different tissues after various time periods by AAS (Table 1). The increase in zinc concentration in the gut 6 hours post exposure followed by the subsequent increase in zinc levels in hemolymph 24 hours post treatment reveals that these NPs were able to cross the gut barrier and reach the hemolymph. From hemolymph they are taken up by the circulating hemocytes as revealed by continuous increase in zinc levels in these cells. The uptake of NPs by hemocytes was also confirmed by EDX analysis (Figs. 2 and 3). As is evident in spectrum as well as elemental mapping (Fig. 3), the zinc ions are present on the hemocytes’ surfaces. The decline in cell viability after ZnO exposure can be attributed to the translocation of these NPs across the gut barrier into the hemolymph and their subsequent interaction and/or uptake by the circulating hemocytes therein.

**Table 1:** Accumulation/Distribution of Zinc Oxide nanoparticles in various tissues.

Time\Tissue	μg/g body weight (or μg/ml in case of hemolymph)					
	Whole Insect	Gut	Hemolymph	Haemocytes	Cuticle	Excreta
0 hours	*	#	#	#	#	#
6 hours	4.064 ± 0.033	1.100 ± 0.083	0.850 ± 0.057	0.110 ± 0.005	0.102 ± 0.009	0.209 ± 0.015
12 hours	3.516 ± 0.039	0.878 ± 0.030	2.128 ± 0.055	0.416 ± 0.020	0.250 ± 0.014	0.298 ± 0.017
24 hours	3.306 ± 0.041	1.025 ± 0.078	1.851 ± 0.058	0.904 ± 0.016	0.694 ± 0.036	0.452 ± 0.022
48 hours	2.426 ± 0.023	0.826 ± 0.031	1.170 ± 0.042	0.878 ± 0.022	0.577 ± 0.029	0.991 ± 0.033
72 hours	2.065 ± 0.032	0.717 ± 0.041	0.945 ± 0.052	0.619 ± 0.019	0.403 ± 0.009	1.057 ± 0.034
5 days	1.824 ± 0.031	0.179 ± 0.024	0.408 ± 0.019	0.185 ± 0.016	0.108 ± 0.012	0.527 ± 0.023

After feeding larvae the leaves charged with ZnO NPs, zinc level was estimated quantitatively by Atomic Absorption Spectroscopy (AAS) in different tissues after specific time periods, and the values (mean ± SEM) are presented as μg of zinc ions per gram of insect tissue dry weight (or μg/ml of hemolymph). *Indicates that the values are below the detection limits of the instrument. ^#^Indicates that the samples were not evaluated as the whole insect tissue sample, in case of control, yielded no signal.

**Figure 4:**
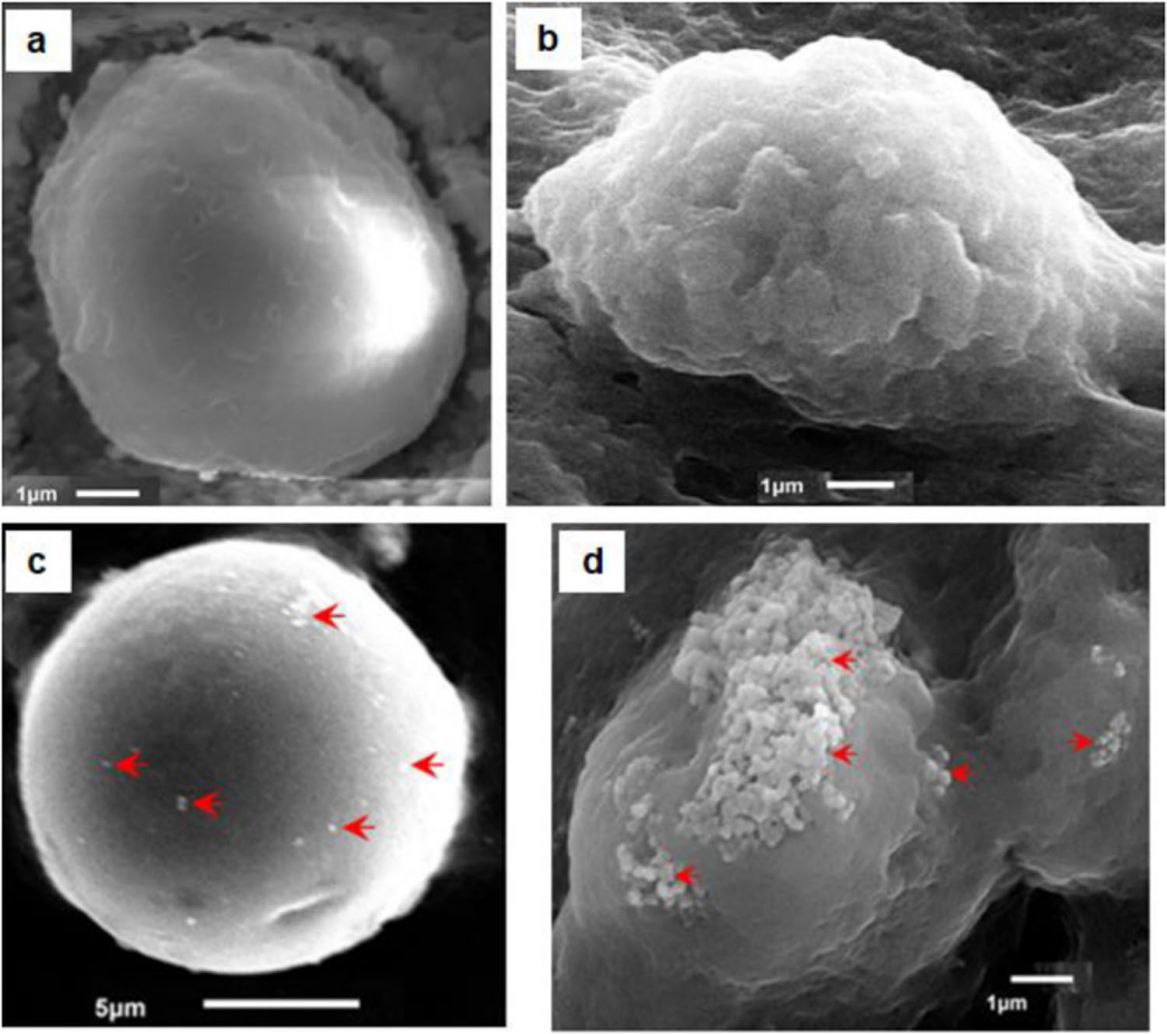
Scanning Electron Micrographs of normal hemocytes (**a,b**) compared with their counterparts 24 h post ZnO NP treatment (**c,d**). The Prohemocytes (**a**) and the Plasmatocytes (**b**) are representatives of the nonphagocytic and phagocytic cell types, respectively. Nonphagocytic hemocytes possibly take NPs through general diffusion across the plasma membrane and are thus involved in limited trafficking (**c**); whereas, the phagocytic cells seem to be involved in the selective/regulated uptake of aggregated NPs (**d**). Red arrows indicate the nanoparticles.

The uptake of NPs is also observed in SEM micrographs of hemocytes. As is evident in Fig. 4, the NPs can be seen on the surface of hemocytes. Two different types of hemocytes have been shown in the micrographs: (1) prohemocyte, a nonphagocytic hemocyte (Fig. 4a) and (2) plasmatocytes, a phagocytic hemocyte (Fig. 4b). The micrographs, possibly, reveal two different modes/processes by which the hemocytes belonging to different lineages are involved in the uptake of these NPs. Prohemocytes, which represent the nonphagocytic cell lineage, are responsible for limited uptake (Fig. 4c); whereas, plasmatocytes, which represent a major phagocytic cell lineage, are responsible for bulk uptake of these NPs possibly by phagocytosis (Fig. 4d).

### ZnO NPs induced morphological changes in hemocytes

Using light microscopy, we were able to identify five hemocyte types in the larval hemolymph namely prohemocytes, plasmatocytes, granulocytes, oenocytoids and spherulocytes (see Supplementary Fig. S2). The hemocytes in control group had well defined morphology and intact structural features. Whereas, ZnO NP exposure resulted in various structural abnormalities like disruption of cell membrane, altered morphology, hypervacoulation, *etc*. (see Supplementary Fig. S3). The morphological alterations in the presence of ZnO NPs coincided with the declines in cell viability and may be, thus, assumed to be associated with inhibition of proliferation and/or cell death.

### ZnO NP exposure increased intracellular ROS

**Figure 5:**
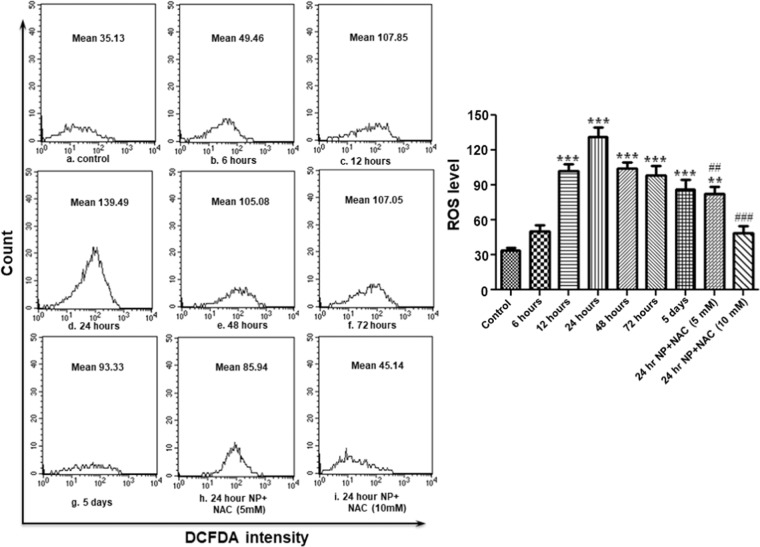
ZnO NP exposure results in ROS (peroxide) generation in *B. mori* hemocytes. After exposing larvae with ZnO NPs for different time periods, hemocytes were collected and stained with DCFH-DA for measurement of peroxide (H_2_O_2_) by flow cytometry. Each experiment was performed at least in triplicate. The larvae were pre-treated with ROS inhibitors (5 mM and 10 mM NAC), and then treated with the nanoparticles. ***P < 0.01, ***P < 0.001 *versus* control. ^##^*P* < 0.01, ^###^*P* < 0.001 *versus* 24 hour group.

**Figure 6:**
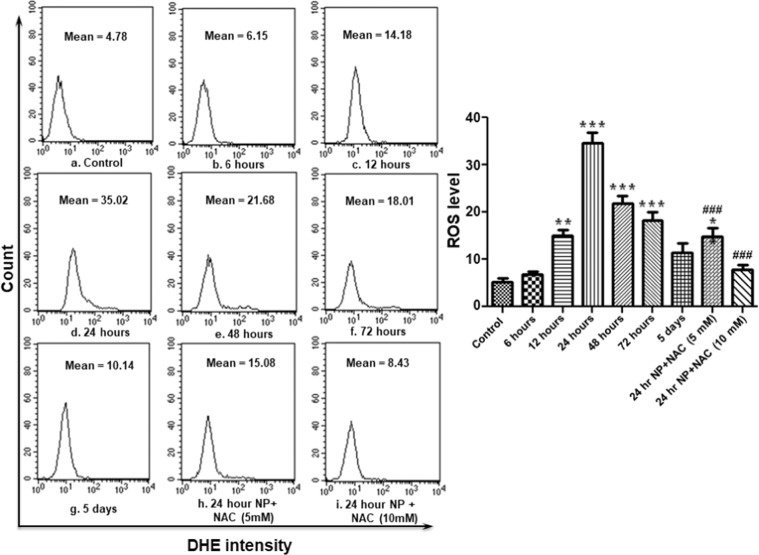
ZnO NP exposure results in ROS (superoxide) generation in *B. mori* hemocytes. After exposing larvae with ZnO NPs for different time periods, hemocytes were collected and stained with DHE for measurement of superoxide (O_2_^−2^) by flow cytometry. Each experiment was performed at least in triplicate. The larvae were pre-treated with ROS inhibitors (5 mM and 10 mM NAC), and then treated with the desired dose of nanoparticles. *P < 0.05, **P < 0.01, ***P < 0.001 *versus* control. ^###^*P* < 0.001 *versus* 24 hour group.

Reactive oxygen species (ROS) are important intermediates in oxidative metabolism. But, their excess generation can damage cells by oxidizing lipids and disrupting structural proteins, enzymes and nucleic acids. By employing DCFH-DA and DHE, cell permeable oxidation-sensitive probes, we observed that ZnO NP exposure resulted in elevated oxidative stress inside hemocytes as revealed by significant increase in peroxide as well as superoxide levels (Figs. 5 and 6). Peroxide levels increased continuously till 24 hours post treatment and thereafter, although, it decreased but was still significantly higher than the control group even 5 days after the exposure. The treatment of NAC (5 mM as well as 10 mM) resulted in significant decline in ROS levels (both superoxide and peroxide).

### ZnO NPs induced apoptosis in larval hemocytes

**Figure 7:**
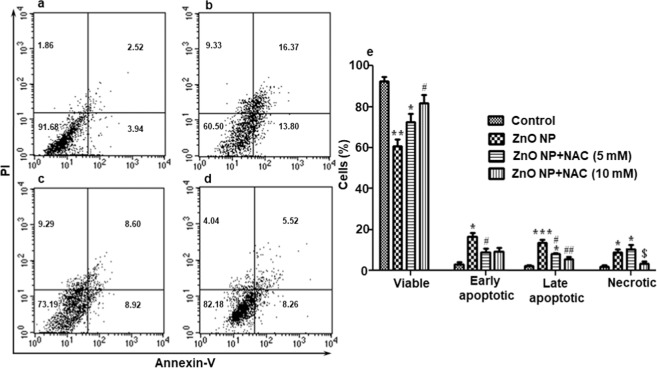
Determination of induction of apoptosis in hemocytes by flow cytometric analysis using Annexin V-PI staining following ZnO NP exposure. (**a–d**) are the representatives of experimental results of one group. Control (**a**), exposed to ZnO NP (**b**), treated with ZnO NPs + 5 mM N-acetyl-L-cysteine (NAC) (**c**) and treated with ZnO NPs + 10 mM N-acetyl-L-cysteine (NAC) (**d**). The graph (**e**) represents the results of the whole experiment (3 replicates), the statistical analysis of which was done by One-way ANOVA test, followed by Tukey’s multiple comparison test. The results in control and vehicle-treated larvae were similar (data not given). *P < 0.05, **P < 0.01, ***P < 0.001 *versus* control. ^#^*P* < 0.05, ^##^*P* < 0.01 *versus* 24 hour group. ^$^*P* < 0.05 *versus* ZnO NPs + 5 mM (NAC).

**Figure 8:**
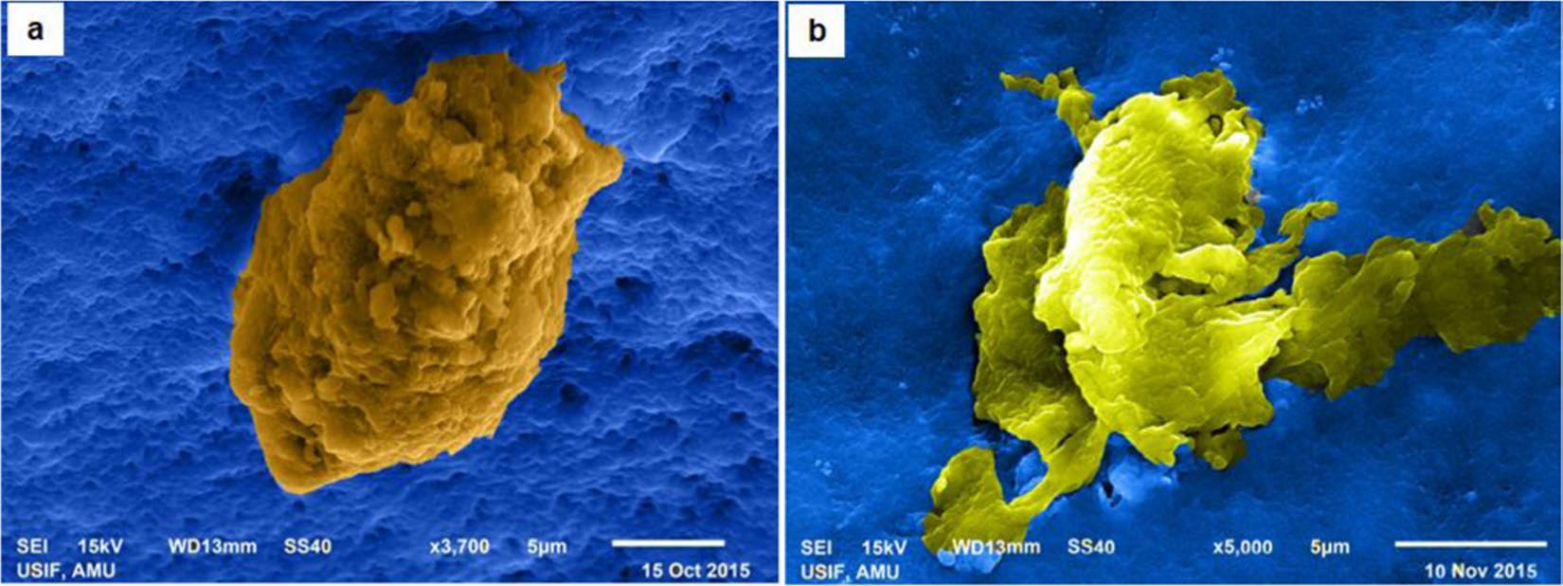
Scanning electronic micrographs of hemocytes undergoing apoptosis. Exposure to ZnO NPs induced apoptosis in the hemocytes of silkworm larvae. These micrographs show the hemocytes caught, possibly, in different stages of apoptosis; the early stage (**a**) characterized by bleb formation and the advanced stage (**b**) marked by the disruption of cell membranes. The micrographs have been artificially colored to enhance their contrast.

ZnO NP exposure led to the induction of apoptosis of these larval hemocytes as revealed by flow cytometric analysis using Annexin-V FITC. There was significant increase in apoptotic cell death as indicated by increase in the percentage of cells in lower right and upper right quadrants (Fig. 7), which represent the percentage of cells in early and late apoptotic stages, respectively. There was also increase in the percentage of necrotic cells, represented by upper left quadrants in Fig. 7. The larvae pre-treated with NAC, before being subjected to NP exposure, showed significant reduction in the percentage of apoptoic as well as necrotic cells (Fig. 7c,d). The evidence of cells undergoing apoptosis following NP exposure was also observed by electron microscopy (Fig. 8). We observed hemocytes in different stages of apoptosis based on their morphological features. The early apoptotic cells were marked by bleb formation (Fig. 8a) and the late apoptotic cells by membrane disruption (Fig. 8b).

### ZnO NPs decreased intracellular GSH content and increased MDA levels

The GSH content significantly declined in cells exposed to ZnO NPs compared with the control group cells (see Supplementary Fig. S4). The decline in GSH reached maximum level 24 hours post exposure and thereafter there was an apparent tendency to return to normal levels.

Malondialdehyde (MDA) is an important marker of cell membrane injury. ZnO NP exposure resulted in continuous increase in MDA levels and reached the levels significant higher than the unexposed group after 12 hours of exposure (see Supplementary Fig. S5). After 24 hours of NP exposure, the MDA reached the maximum level. It then declined continuously and after 5 days of exposure reached the levels that were not significantly higher than the levels in control group.

### ZnO exposure resulted in alterations in hemocyte dynamics

The exposure of ZnO NPs resulted in change in DHC and THC. The THC increased immediately after NP exposure (see Supplementary Fig. S6) and reached the highest value after 12 hours. Thereafter, it declined continuously and reached the lowest number at 48^th^ hour of exposure, and then showed a tendency to shift towards normal number.

There was significant increase in granulocyte population after 12 and 24 hours of NP exposure; whereas, a significant increase in plasmatocytes was observed post 12 hour NP exposure. Other changes observed in DHC were decrease in the population of prohemocytes and spherulocytes 12 hours post NP treatment (see Supplementary Fig. S7).

## Discussion

We undertook this study with the aim to investigate the accumulation of one of the most commonly used NPs, i.e., ZnO NPs in an invertebrate model system, *B. mori*, following a single oral exposure and the resulting effects on its hemocytes, which constitute the mainstay of invertebrate innate immune system.

ZnO NPs are among the most widely used nanomaterial and the large-scale use will inevitably lead their way to the environment from their synthesis, usage, and disposal. Therefore, there are widespread apprehensions with regard to their fate and impacts on life and environment.

The digestive track is the most likely route of NP entry, for direct exposure through intentional ingestion as well as for indirect exposure by means of their dissolution from food containers or by secondary ingestion of inhaled particles^43^. In addition to this, the widespread utilisation of these NPs may result in environmental contamination and thus unintentional exposure via food, water, *etc*. In the present study, the NPs were administered via food which is supposed to be one of the natural routes through which animals may be exposed to commonly used engineered NPs. This administration route takes care of the degradation and absorption, common in oral exposure, of NPs by various biological barriers like digestive tract cells. Thus, uptake and trafficking of NPs and their impact on immune-competent cells (and therefore innate immune system) can be determined following the exposure which closely mimics the manner various organisms are exposed in the natural environment. Fourth instar larvae (at 48 h after ecdysis) were selected, as the insects at this stage can be easily observed, handled, manipulated, enough test sample can be obtained and damage to the larvae is reduced/minimal^44^.

First of all, the accumulation of ZnO NPs was determined in various tissues after different time periods to have a clue about the possible uptake and clearance from the insect system. This enabled us to determine the time point when NPs, after crossing the gut barrier, reach the immune-competent cells in the hemolymph. Further, the first step towards understanding the fate and effects of NPs would be to visualize nanoparticle presence and interactions with the cell membrane of various cells, i.e., whether they physically interact with the membrane systems or not. For this, the employment of SEM coupled with EDX evinced the physical interaction of these NPs.

Our results indicate that ZnO NPs have the potential to cross insect gut barrier. The propensity of NPs to cross the biological barriers is alarming because of the risks posed by their non-intended uptake. To reach inside the body of organisms, these NPs have to face various cellular as well as acellular barriers. For example, mucus forms an effective acellular barrier for, almost, the entire GI tract. Previously, it was thought that nanomaterials are not capable to penetrate the mucus layer. But these assumptions have been sidelined in view of considerable evidence against it^45,46^, and, in fact, NPs have been shown to change the composition and secretion of mucus itself^47,48^. Additionally, the extracellular matrix (ECM) and the pericellular matrix (PCM) surrounding the cell may influence the interactions of NPs and various biological barriers. The interaction of NPs with the complex structure of the ECM is now well established^49,50^. It has been postulated that the filtering effect of the ECM (due to its dense structure) and its distinct charge properties may hinder the passage of large-sized NPs but, at the same time, the movement of very small NPs (less than about 20 nm) may not be hampered and may, thus, exhibit higher toxic potential^51^. The type of interaction of NP with cell membrane may ultimately decide the intracellular trafficking, localization and compartmentalization, cellular retention, *etc*. of the NP in question^52^, *e.g*., surface modified NPs were reported to have five-fold greater affinity and get readily internalized than their unmodified versions^53^. Additionally, the cationic NPs are reported to penetrate the cellular membranes rapidly as compared to the anionic counterparts possibly because they disintegrate the surface feature and generate hydrophilic pores in the bilayer^54^.

The NPs can cross the biological barriers either by transcellular passage (i.e., through the cells) or paracellular passage (i.e., between the cells). Because of the lipophilic composition of plasma membranes, the transcellular route is preferred for passive uptake of lipophilic substances while hydrophilic substances are usually taken up by paracellular route. The paracellular route, because of extremely small penetration area, is restricted for the passage of polar substances below 1000 D. Therefore, the NPs, being larger than 1000 D, are not expected to employ paracellular route^55^. But, Geiser *et al*.^56^ showed that even passive transcellular diffusion is responsible for the passage of TiO_2_ NPs (22 nm) through the cells.

As of now, the popular viewpoint is that endocytosis is the most likely mode of NP uptake (both metal and metal oxide NPs). The main routes of endocytotic uptake of NPs include macropinocytosis, clathrin-mediated endocytosis and caveolae-dependent endocytosis, with clathrin-mediated endocytosis appearing to be the main route of uptake of modified as well as unmodified NPs^57–62^. Based on our findings, it can be suggested that different cell types uptake NPs through different routes. For instance, non-phagocytic cells (like prohemocytes) pick up NPs by simple diffusion and are thus involved in limited uptake of these NPs whereas the phagocytic cells (granulocytes and plasmatocytes) do this through regulated processes, presumably endocytosis, and are, therefore, involved in the bulk uptake of these NPs. Further, this might not be reflecting the entire picture and there is every possibility that the interactions and uptake depend on the type of NP and its physico-chemical properties, cell type, route of exposure, *etc*.

**Figure 9:**
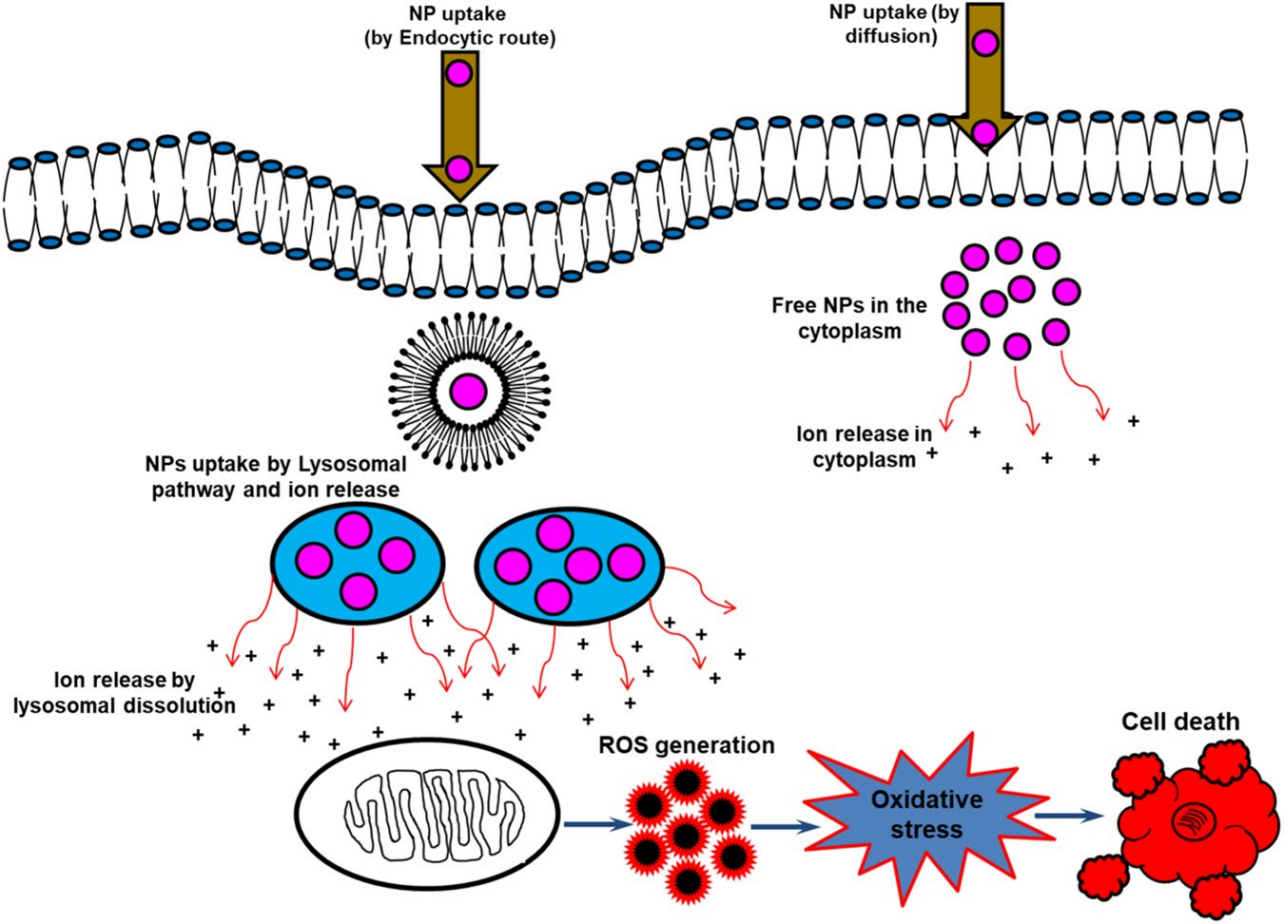
Schematic of the uptake and the general toxicity mechanism of ZnO NPs. The NPs may be taken up by the cells by active internalization or endocytosis-free mechanisms (the uptake may also dependent on the cell type involved). NP uptake by energy-dependent processes are confined in vesicular structures, endosomes, and finally in lysosomes. The acidic lysosomal pH may trigger enhanced release of the relatively toxic ions, which may subsequently result in elevated ROS levels thereby inducing apoptosis, DNA and membrane damage, and other toxic responses. The limited uptake and ion release by endocytosis-free mechanisms may be responsible for the manifestation of only a small part of the overall toxic responses (modified from Sabella *et al*., 2014, Published by The Royal Society of Chemistry^76^).

The NPs, after reaching the hemolymph and interacting with hemocytes, caused various toxic responses (Fig. 9) such as they resulted in significant decline in hemocyte viability, generated significant ROS, caused various morphological alterations, induced apoptotic cell death, *etc*. These effects may be attributed either to higher catalytic properties (due to size and/or surface modifications), due to which they interfere with various biochemical processes, or to leakage of ions following decomposition of these NPs, which disrupts the intracellular homeostasis of free metal ions. The increase in toxic responses with the increase in the concentration of zinc in hemolymph and hemocytes implies that that the toxic responses are mainly due to abnormal increase in Zn^2+^, which ultimately leads to the elevation of ROS level and decline in cell viability. We also observed a significant decline in GSH content in cells exposed to ZnO NPs and its depletion coincided with the enlarging tendency of intracellular ROS level demonstrating that ZnO NPs damaged the antioxidant mechanism of these immune surveillance cells and a significant increase in MDA levels indicated the potential of these NPs to cause lipid peroxidation. Furthermore, we observed, after an initial peak, a decline in ROS generation, restoration of cell viability, decrease in lipid peroxidation, *etc*. continuing up to the 5^th^ day of exposure. This trend correlated with the continuous decline of these NPs in various tissues and their subsequent excretion from the body. Therefore, it can be assumed that the toxic responses of these NPs are due to their interactions and/or uptake by the hemocytes inside the insect’s body, and as the insects get rid of the NP load, by continuous secretion of these NPs from their body, the toxic responses diminish with time.

These observations are consistent with earlier reports wherein NP exposure has been implicated in similar types of effects, both *in vivo* as well as *in vitro*. Generally, the small size and large surface area of NPs is presumed to produce ROS and oxidative stress^63^, and this resulting oxidative stress has been inculpated for the observed toxicity of these NPs. Various means have been proposed for NP induced ROS generation^64,65^. The NPs, owing to their unique chemical and surface characteristics, can lead to spontaneous ROS generation at their surface, or their specific interactions with various cellular components and their interventions in cell signalling pathways may also lead to the generation of free radicals. Activation of enzyme NADPH-oxidase, responsible for the production of O^2−^ in the membrane of phagocytes, can be yet another source of ROS.

ROS generation, in case of ZnO NPs, has been attributed to their semiconductor as well as nano-level characteristic, and this leads to ROS generation even in the absence of light. It has been reported that the ZnO nanocrystal quality decreases with size resulting in elevated interstitial zinc ions and oxygen vacancies^66,67^. These crystal defects have been shown to lead to a large number of electron-hole pairs that have the potential to migrate to the NP surface and, thus, contribute to the generation of ROS. The presence of oxygen and hydroxyl ions in the aqueous environment of ZnO NPs can react with these electrons and holes, respectively. This process generates free radicals that are highly reactive such as the superoxide anions (from electrons) and the hydroxyl ions (from holes)^63^. These radicals, when in contact with the cellular environment, can result in the oxidation and reduction of various macromolecules including DNA, lipids and proteins; hence, leaving cells vulnerable to significant oxidative damage.

Apart from this, NPs, because of their small size, are thought to be capable of reaching the nucleus and interacting with DNA^67–69^, and their ability to generate ROS may also lead to the manifestation of indirect effects on DNA^67^. This DNA damage may disrupt normal cell functions, lead to carcinogenesis and/or cell death. We observed the potential of these NPs to reduce the cell viability and induce apoptotic cell death. Further, pre-treatment with NAC resulted in significant decline in ROS which subsequently led to the decrease in the percentage of cells undergoing apoptosis. This revealed the potential of these NPs to induce ROS mediated apoptosis. The elevated level of ROS may result in the induction of other damages because they have the potential to affect various cells and their normal functioning. Further, the continuous exposure to various NPs may result in other complications or aggravate the already existing ones because ROS and oxidative stress has been linked with various age-related degenerative diseases^70–73^.

Additionally, exposure to ZnO NPs was observed to alter the hemocyte dynamics in silkworm larvae. There was an increase in THC immediately post NP exposure and this increase might be the response to perceived immune challenge as these NPs crossed the gut barrier and reached the hemolymph. This increase in THC might be due to the release of these hemocytes either from enhanced rate of cell divisions or from attached hemocyte populations because, in the Lepidopteran larvae, the maintenance of circulating hemocytes has been ascribed to their mitosis in the circulatory system as well as their release from various hematopoietic organs^74^. We observed a decline in prohemocyte proportion immediately after NP exposure, reaching the lowest level 12 hours post exposure and thereafter its population shifted towards the normal levels. On the other hand, there was a significant increase in the proportion of granulocytes and plasmatocytes, and, after an initial peak (post 12 and 24-hour exposure), their proportions plateaued around the normal levels. To explain these observations, it can be stated that the immediate response to NP challenge was the differentiation of prohemocytes into the phagocytes. This lowered the percentage of prohemocytes and increased that of the two professional phagocytes, *i.e*., granulocytes and plasmatocytes. Insect hemocytes are a complex of cell types that have the ability to recognize and phagocytose the foreign bodies in the hemolymph. Phagocytosis is an important cellular immune response responsible for removing invading bacteria and other minute foreign entities^75^. Plasmatocytes and granulocytes are two major cell types involved in this process of phagocytosis. The tendency of the THC as well as DHC to reach the normal levels indicates that the presence of NPs within insect body is responsible for these responses and as the insects rid themselves of the NP load, by the continuous elimination of these NPs from their system, they return to normal physiological states and there is restoration and repair of various systems and their affected pathways.

In summary, our experiments showed that the NPs have the potential to cross biological barriers and induce various toxic effects, which are mainly due to their ROS generating potential. It is also noteworthy to mention here that NP exposure might have resulted in hematopoiesis impairment and their subsequent clearance from the insect system might have resulted in the process of growth and recovery of hematopoiesis in the hematopoietic organs resulting in the decline of elevated ROS to normal level. However, it is not certain whether these NPs are first taken up as such by the cells (by diffusion or by phagocytosis) and their corresponding ions released inside these cells, or their ions are first released in the hemolymph and then taken up by the hemocytes. It is further suggested that the *in vivo* mechanism/action of these NPs may differ from the *in vitro* mechanism/action, from one organism to another, or even between various cell types. Therefore, further research is required in this direction that should characterize the nature and interactions of various NPs inside these cells. Nanoparticles, being an attractive component of many applications, necessitate the understanding of their biological interactions and the assessment of their benefits as well as associated risks.

## Supplementary information

Supplementary information.
