# Supplementary material for: Accumulation and trafficking of zinc oxide nanoparticles in an invertebrate model, Bombyx mori, with insights on their effects on immuno-competent cells
Source: Sci Rep. 2020 Jan 31;10:1617. doi: 10.1038/s41598-020-58526-1 (PMC6994675; doi:10.1038/s41598-020-58526-1)
Supplement: Supplementary file 1 — Supplementary information. [file 41598_2020_58526_MOESM1_ESM.pdf]

Accumulation and trafficking of zinc oxide nanoparticles in an invertebrate model, *Bombyx mori*, with insights on their effects on immuno-competent cells

Ashiq Hussain Mir[1,3], Ayesha Qamar[1], Ishana Qadir[1], Alim H Naqvi[2], Rizwana Begum[1]

[1]Section of Entomology, Department of Zoology, Aligarh Muslim University, Aligarh-202002, Uttar Pradesh, India

[2]Interdisciplinary Nanotechnology Centre, Aligarh Muslim University, Aligarh-202002, Uttar Pradesh, India

[3]Department of Zoology, School of Bioengineering and Biosciences, Lovely Professional University, Phagwara-144411, Punjab, India

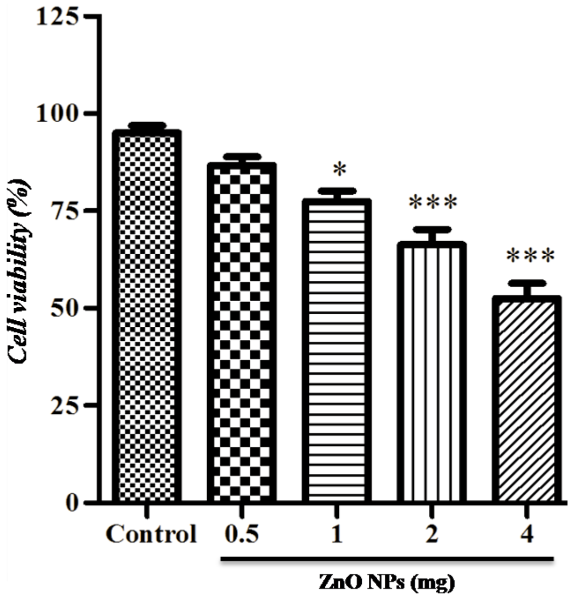

Fig. S1: Effects of different concentrations of ZnO NPs on viability of *B. mori* haemocytes as demonstrated by trypan blue exclusion assay. Data are expressed as mean ± SEM, with *n=3* per group. *P<0.05, ***p <0.001 *versus* the control group.

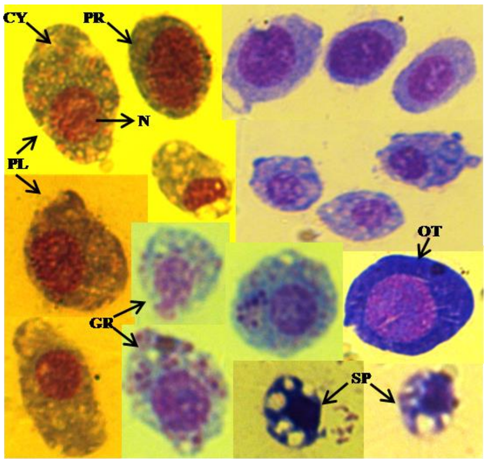

Fig. S2: Light microscopic images of different types of hemocytes in *B. mori* (magnification x400). The hemocytes were identified on the basis of size, morphology, nucleus to cytoplasm ratio, presence or absence of granules, etc. PR= prohemocytes, PL= Plasmatocytes, GR= Granulocytes, SP= Spherulocytes, OT= Oenocytoids, N= Nucleus, CY= Cytoplasm

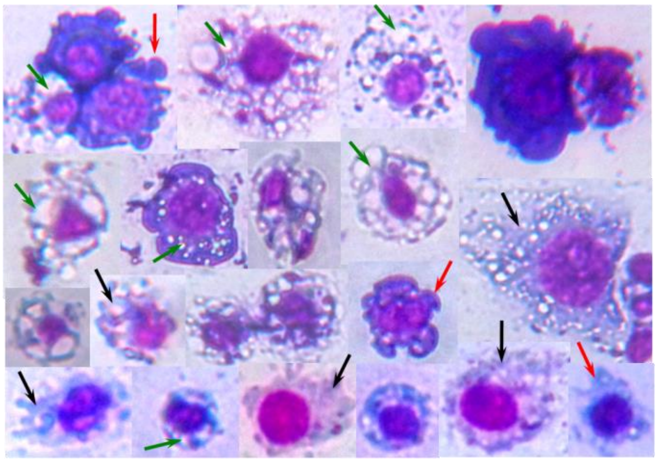

Fig. S3: Light microscopic images of *B. mori* hemocyte subpopulations (magnification x400) after ZnO NP exposure. Various types of damage caused by NP exposure are shown in this plate. Black arrows indicate cell membrane disruption, red arrows alteration in cell shape including bleb formation and green arrows hypervacuolation.

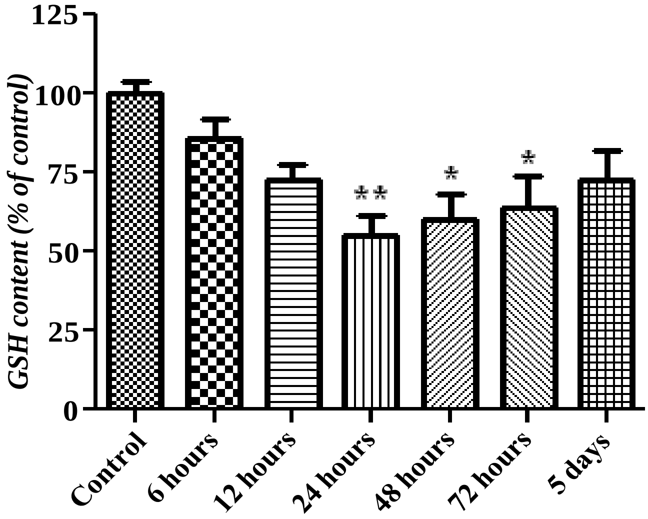

Fig. S4. ZnO NP exposure led to decline in GSH content in *B. mori* hemocyte. After exposing larvae with ZnO NPs for different time periods, there GSH content was estimated. **P* < 0.05, ***P* < 0.01 versus control.

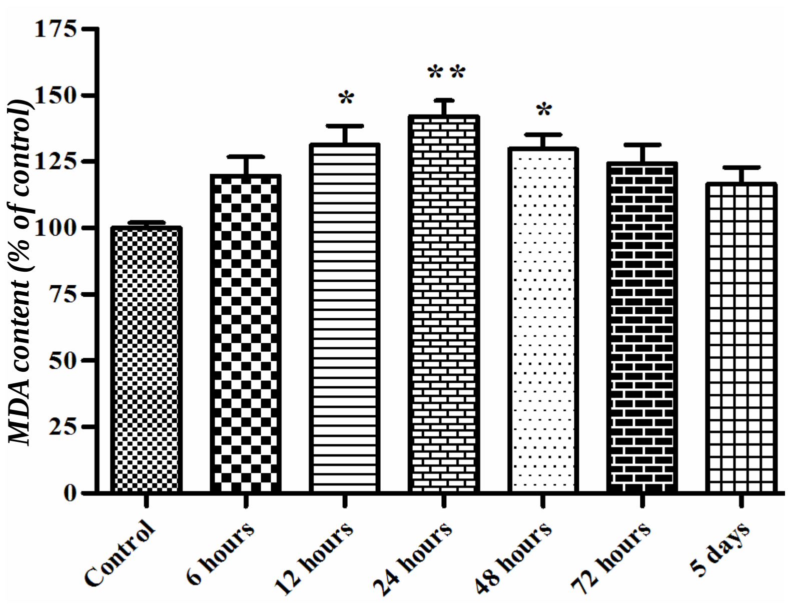

Fig. S5. Cellular MDA content in *B. mori* hemocyte after ZnO NP exposure. After exposing larvae with ZnO NPs for different time periods, their MDA content was estimated. *$P < 0.05$, **$P < 0.01$versus control.

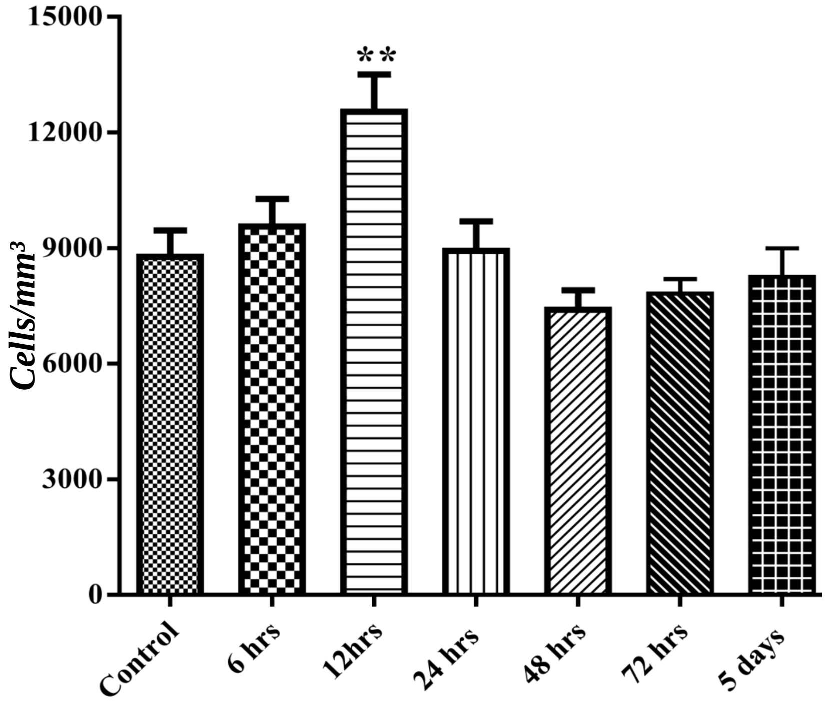

Fig. S6. ZnO NP exposure resulted in change in THC in *B. mori* hemocyte. After exposing larvae to ZnO NPs for different time periods, their THC was estimated; **$P < 0.01$versus control.

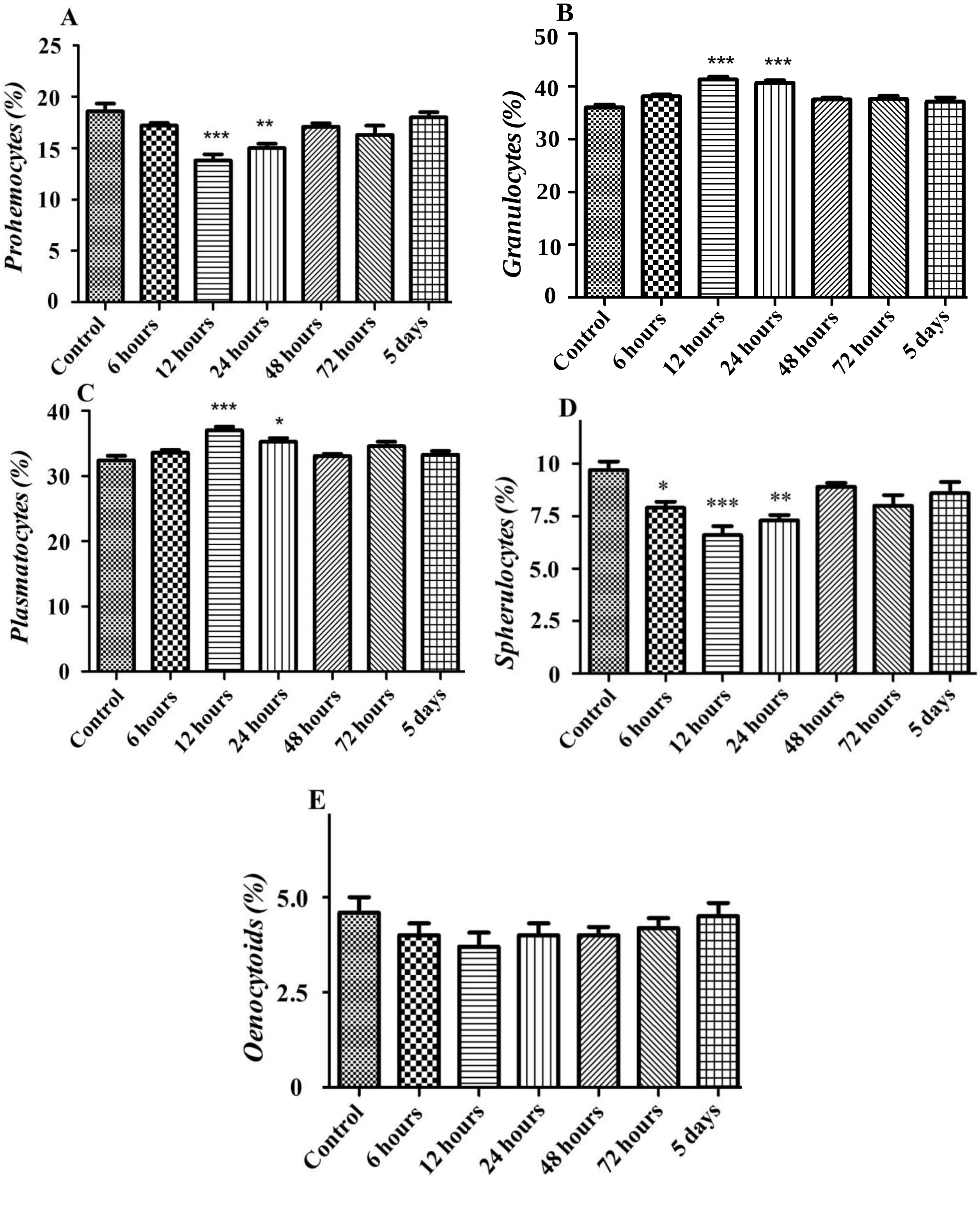

Fig. S7. ZnO NP exposure resulted in change in differential hemocyte count (DHC) in *B. mori* hemocytes. After exposing silkworm larvae to ZnO NPs for different time periods, their DHC was estimated. A) prohemocytes, B) granulocytes, C) plasmatocytes, D) Spherulocytes, and E) oenocytoids. $*P < 0.05$, $**P < 0.01$, $***P < 0.001$ versus control.
